# Dynamically Stable Topological Phase of Arsenene

**DOI:** 10.1038/s41598-019-44444-4

**Published:** 2019-05-28

**Authors:** Gul Rahman, Asad Mahmood, Víctor M. García-Suárez

**Affiliations:** 1 Department of Physics, Quaid-i-Azam University, Islamabad, 45320 Pakistan; 2 Departamento de Física, Universidad de Oviedo, 33007 Oviedo, Spain; 3 Nanomaterials and Nanotechnology Research Center (CINN), CSIC - Universidad de Oviedo, El Entrego, 33940 Spain

**Keywords:** Two-dimensional materials, Two-dimensional materials, Two-dimensional materials, Two-dimensional materials

## Abstract

First-principles calculations based on density functional theory (DFT) are used to investigate the electronic structures and topological phase transition of arsenene under tensile and compressive strains. Buckling in arsenene strongly depends on compressive/tensile strain. The phonons band structures reveal that arsenene is dynamically stable up to 18% tensile strain and the frequency gap between the optical and acoustic branches decreases with strain. The electronic band structures show the direct bandgap decreases with tensile strain and then closes at 13% strain followed by band inversion. With spin-orbit coupling (SOC), the 14% strain-assisted topological insulator phase of arsenene is mainly governed by the *p*-orbitals. The SOC calculated bandgap is about 43 meV. No imaginary frequency in the phonons is observed in the topological phase of arsenene. The dynamically stable topological phase is accessed through *Z*_2_ topological invariant *ν* using the analysis of the parity of the wave functions at the time-reversal invariant momentum points. The calculated *ν* is shown to be 1, implying that arsenene is a topological insulator which can be a candidate material for nanoelectronic devices.

## Introduction

Since the discovery of graphene^1–4^, much attention has been devoted to discover new two-dimensional (2D) materials due to their exceptional properties such as high electrical conductivity, mechanical strength, band tunability etc. Graphene, which started as the most popular material among the scientific community, has lost however some ground due to its lack of intrinsic band gap, which limits its applications in electronic devices. New 2-D elemental materials with a band-gap such as silicene, germanene, stanene, phosphorene, arsenene, etc.^5–12^ have nevertheless been discovered in the last decade. Most of these 2D materials are direct bandgap materials with a bandgap of less than 2.0 eV, which further limits their use in optoelectronic devices. A suitable 2D material with a direct bandgap for optoelectronic applications is arsenene -a monolayer of As atoms–, which was proposed by Kamal and Ezawa^12^ in 2015. This material can be obtained from the bulk form of arsenic, which has a layered crystal structure –gray arsenic, which is a semi-metal^13^. Kamal and Ezawa^12^ studied a monolayer of arsenic, with either a planar, buckled, or puckered structure, and concluded that buckled arsenene is more stable than puckered and planar arsenene. The electronic structure of buckled arsenene with a buckling parameter Δ = 1.39 Å corresponds to that of a semiconductor with an indirect/direct bandgap of 1.63/1.97 eV^12^. Since then much attention has been diverted to arsenene to explore its physical properties under different conditions such as strain or electric fields. For instance, semimetal–semiconductor and indirect–direct band gap transitions can be driven by applying biaxial strain and electric fields perpendicular to the plane of arsenene^14^. This material can also be passivated with hydrogens (hydrogenated arsenene) and becomes quasi-planar with a magnetic ground state^15^.

Some two dimensional materials also behave as topological insulators (TIs). These materials, which were observed for the first time in HgTe quantum wells^16^ and are also known as quantum spin Hall (QSH) insulators, are novel nonmagnetic insulators, and are currently attracting intense worldwide interest^17–22^. A unique property of a QSH insulator is the presence of conducting edge states, which carry two counter-propagating spin-polarized currents. Due to constraints of time-reversal symmetry, these conducting edge states hinder backscattering, making them very desirable for spintronics and other applications. Although graphene was initially proposed to be a host for QSH states, the spin-orbit coupling (SOC) in this material is very weak, and the associated gap is too small to be accessible experimentally^23^. This, however, motivated the community to explore new 2D TIs and in the last few years many efforts have been made to propose new 2D-TI with strong SOC^24–31^.

In many cases, the TI state in a 2D material can be induced by either external strain or an electric field^29–32^. However, when external strain is applied to a material, the strain energy can either permanently deform the material or bring it to a new phases by inducing soft-modes in phonons. Strain, however, is considered to be a key ingredient to induce topological phases in 2D materials^29–31,33–35^, but the dynamic stability of the TI state has been largely ignored in the past. When a 2D material is grown on a substrate, however, the role of compressive/tensile strains cannot be ruled out. Hence, it is necessary to investigate the dynamic stability of arsenene under strain and check whether the strain driven topological state is stable or not. The main purpose of the present work is to address the dynamic stability of arsenene under strain and to investigate whether the strain driven TI phase of arsenene is dynamically stable or not. We note that this last point, which is essential to determine the feasibility of applications based on this material, has not been addressed in many previous studies of TI or, in general, in studies of electronic phase transitions.To address this technologically important issue, we used first-principles calculations based on density functional theory (DFT) to show that strain can induce a TI state in arsenene. The phonon calculations demonstrate that the strain driven TI state is dynamically stable, implying that arsenene can be a candidate material for QSH devices.

## Results and Discussions

Generally, a buckled configuration is always expected to sustain larger tensile strains than a planar structure. We, therefore, studied first the energetic evolution under biaxial tensile/compression strains. In our calculations, the biaxial tensile strain was applied by fixing the lattice constant to a series of values larger than that of the equilibrium state and optimizing the atomic coordinates for each case. To avoid local minima and clearly determine the optimized buckling Δ parameter of arsenene, we also carried out total energy calculations as a function of Δ for different lattice constants *a*. The calculated cohesive energy *E*_*c*_ as a function of Δ is shown in Fig. S1(a). The optimized Δ and *E*_*c*_ for each lattice constant are summarized in Table S1. From these results it is clear that the optimized Δ decreases with increasing lattice constant. By considering Δ as a perturbation, it can be seen that positive (negative) curvatures of *E* vs Δ show stable (unstable) structures. It is then obvious from Fig. S1(a) that planar arsenene has a negative curvature —indicating that planar arsenene is not a stable phase of As. However, as the bucking is introduced, arsenene goes towards a stable buckled state at lattice constants ≥3.61 Å. At larger lattice constants, e.g., 4.37 Å (see Fig. S1(b)), the buckling introduces some metastable and unstable states. This implies that these metastable/unstable states can be washed out when the lattice constant is decreased. From these calculations it is possible then to infer that each lattice constant has its own buckling parameter, which will have a strong influence on the physical properties of each case. Hence, it is expected that arsenene will behave differently when grown on different substrates, since the substrate can change the most stable lattice constant and induce either tensile or compressive strain, which will further stabilize or destabilize it. Table S1 shows that the minimum cohesive energy (−2.94 eV) is achieved for 3.61 Å with a buckling of 1.39 Å. Note that these values agree with previous literature^12,13,15,36,37^.

**Figure 1:**
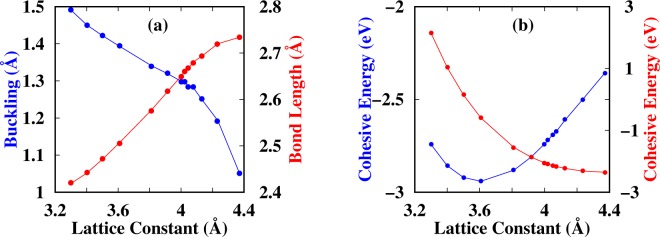
(**a**) Optimized buckling/bond-length (in Å) vs lattice constant (in Å). The blue (red) line represents the buckling (bond length). (**b**) Optimized lattice constants (in Å) vs cohesive energy (in eV) of buckled (left, blue color) and planar (right, red color) arsenene.

Figure 1(a) shows that the bond length (buckling) increases (decreases) as the lattice constant increases. On the other hand Fig. 1(b) shows that the binding energy decreases as the lattice constant increases, with the minimum binding energy found at 3.61 Å and Δ = 1.39 Å. The increase in the buckling and binding energy is mainly due to the increase in the strain energy: compressed arsenene is under compressive strain and to release the excess strain energy, arsenene prefers the buckled structure as compared with the planar one. In the same figure we also show the binding energy of planar arsenene, where it is clear that such configuration is not stable at smaller lattice constants. However, at larger lattice constants (~≥4.0 Å) the planar structure becomes energetically stable (due its negative binding energy). Hence, we speculate that the type of stable structure of arsenene will strongly depend on the substrate, since it determines the amount of strain.

**Figure 2:**
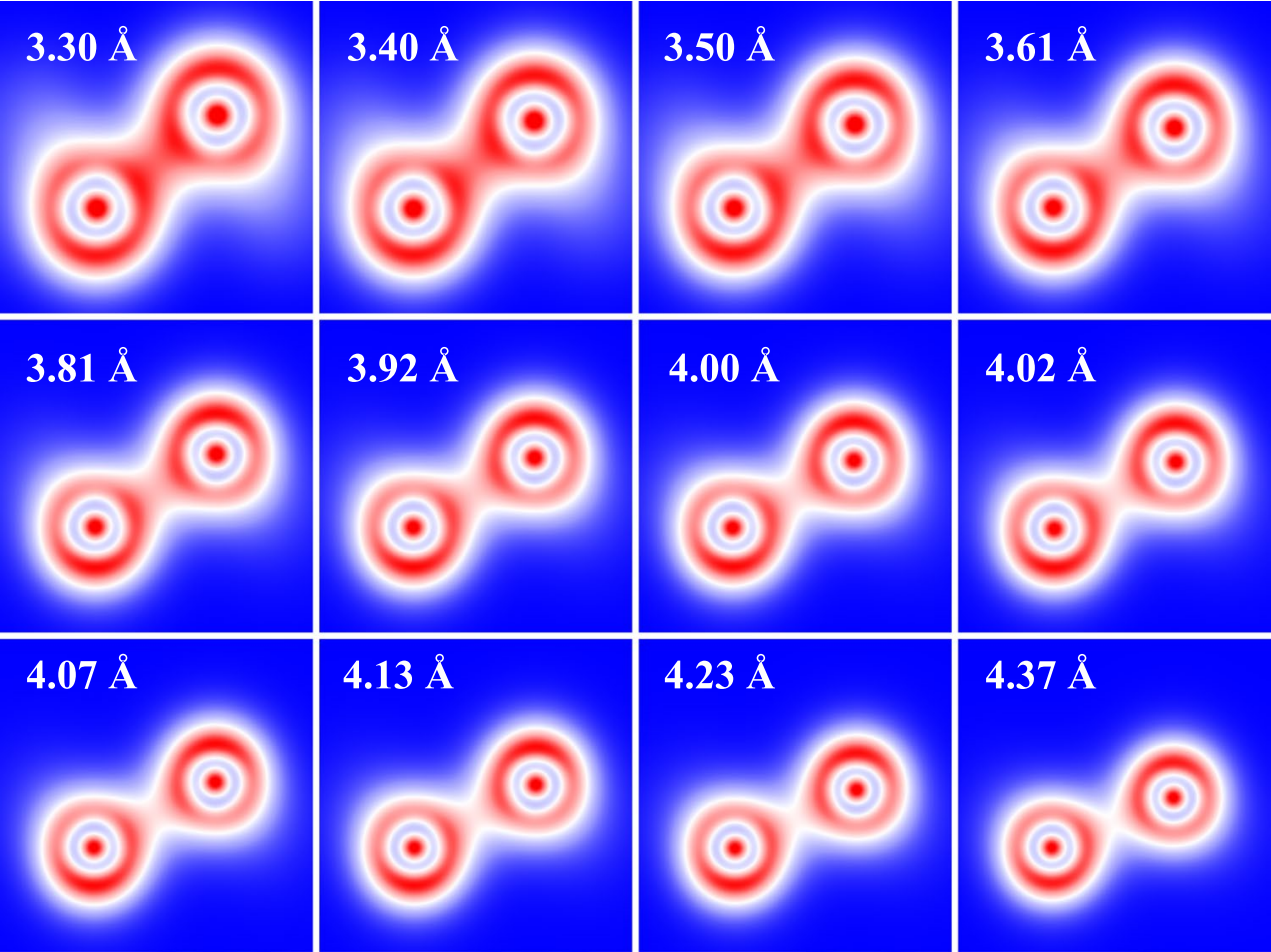
Calculated charge density of arsenene at different lattice constants. The lattice constants are mentioned in the figure, and the optimized Δ can be seen in Table S1.

The bonding between the arsenic atoms in arsenene also changes when it is subjected to tensile/compressive strains. Figure 2 shows the calculated charge density at the optimized *a* and Δ. It is clear that there is a strong covalent bond between the As atoms at smaller lattice constants, and the covalent character is mainly dominated by the *p* electrons. However, as the lattice constant increases, the covalent character decreases and the charges localize at the As atoms. Such localization is expected to decrease the electrical conductivity of arsenene. A detailed charge analysis shows that the charge transfer (from *p* to *s*) increases with increasing *a*. The evolution of charges under strain will then have an effect on the force constants and therefore on the phonons, as will be discussed below.

**Figure 3:**
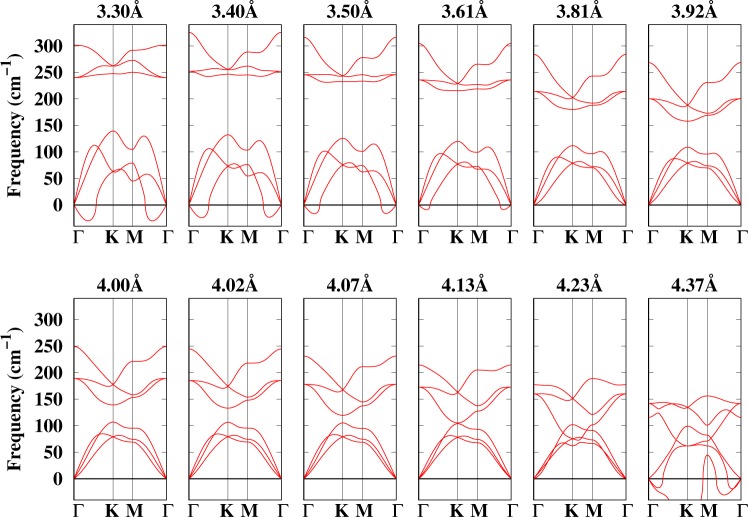
Calculated phonon dispersion curves of buckled arsenene under different strains.

Before we proceed to investigate the electronic properties of arsenene under different strains, it is essential to investigate the dynamic stability of arsenene under strain, which can be done by either using phonon calculations or molecular dynamics. In our case, we calculated the phonon dispersion curves of arsenene under different strains (lattice constants) to look for possible imaginary frequencies that signal the presence of instabilities or structural transitions. Generally, in a lattice with a basis of two atoms in the primitive cell (as we have in arsenene), there are two upper branches of the phonons, known as longitudinal optical (LO) and transverse optical (TO), and two lower branches, known as longitudinal acoustical (LA) and transverse acoustical (TA). Acoustic phonons have frequencies that become smaller at long wavelengths, and correspond to sound waves in the lattice. The artificially generated distance of ~15 Å between the two sheets displaces the atoms in the *z*-direction, which generates out of plane transverse acoustic (ZA) and optical (ZO) frequencies, respectively. Thus six phonon branches are expected in arsenene. Figure 3 shows the calculated phonons for different lattice constants (strains). The TA and LA modes exhibit linear dispersions around the Γ point. Notice that an out-of-plane flexural mode (ZA) can also be seen. Flexural modes, which have been characterized in other 2D materials such as graphene^38–40^, silicene^24,41^, hexagonal boron nitride^42^, molybdenum disulphide^43^, puckered arsenic^44^, and ultrathin silicon membranes^45,46^, have a major role in contributing to the thermal conductivity of 2D materials, both as carriers and as scatterers^47,48^.

Figure 3 shows that at a small lattice constant of 3.30 Å, arsenene has soft modes at the Γ-point (mainly ZA modes). These modes decrease with increasing lattice constant, and are washed out when the lattice constant is larger than 3.61 Å (the equilibrium lattice constant). The washing out of the negative ZA modes is due to the absence of a large buckling for large lattice constants. The presence of negative frequencies involves a dynamic instability in the structure —implying that it would not be possible to synthesise a free standing monolayer of As unless a substrate or some external strain is used to stabilize it. Even at the equilibrium geometry (*a* = 3.61 Å, Δ = 1.39 Å) arsenene has negative frequencies at the Γ-point which implies that, though the structure is energetically stable, it is dynamically not stable —hence a free standing monolayer of arsenene is not possible. We must note that at 3.61 Å all the three modes of the optical branch are Raman active. They are 236 cm^−1^ (doubly degenerate) and 305 cm^−1^ at the Γ-point. These values are in agreement with previous DFT works^12^. To stabilize arsenene some external tensile strain is then required. As the lattice increases the strain energy reduces the buckling, which further stabilises the ZA modes. Therefore, at least a 5% tensile strain would be needed to stabilize the structure, since for such strain all modes have positive frequencies in their respective BZ. The optical and acoustical branches of arsenene are well separated by a gap and the branches associated with the modes of specific atoms overlap with the acoustical modes as the lattice constant increases. Notice that the optical (ZO) and acoustic (TO) branches touch each other at the *K*-point, exactly at 4.13 Å. The overlapping of these two modes at the *K*-point implies a possible phase transition which may occur at large strain. Finally, at very large strains, i.e. 21% (4.37 Å), buckled arsenene with a small buckling (1.05 Å) shows again negative frequencies, which demonstrates that the structure is only stable for lattice constants ≥3.61 Å and ≤4.23 Å. Hence, it can be concluded that arsenene remains stable under ~18% tensile strain. For unstrained arsenene (at 3.61 Å) the obtained optical phonon frequencies are five times smaller than in graphene^49^ (1580 cm^−1^), and 1.8 times smaller than in silicene^50^ (550 cm^−1^). This is due to the smaller force constant and weaker As-As bonds, as compared to the C-C and Si-Si bonds. Graphene also shows common features in the Raman spectra called *G* and *D* peaks^49^, around 1580 cm^−1^ and 1360 cm^−1^. The *G* peak corresponds to the *E*_2*g*_ (C-C stretching mode) phonon at the Γ-point of the Brillouin zone and the *D* peak, which is activated by structural defects, arises from the TO phonon mode near the *K*-point in the Brillouin zone^51^. These points are also calculated for buckled arsenene as a function of the lattice constant (strain). The data, summarized in Table S1, show that both *G* and *D* decrease with increasing lattice constant. The decrease in *D* and *G* can be attributed to the weakening of the As-As bonds as the strain is increased, which is consistent with the analysis of Fig. 1. As arsenene is strained, the *G* value decreases, implying that the *sp*^2^ hybridization decreases (the *G* band is due to the *sp*^2^ hybridization). The *D* band is mainly due to disorder in the system (deviation from the planar geometry), so the decrease of *D* with strain implies that the buckling in the system decreases. With the knowledge of *G* for arsenene under biaxial strain, we can calculate the Grüeinsen parameter, which is given by $\gamma =-\,{\rm{\Delta }}{\omega }_{G}/2{\omega }_{G}^{0}\varepsilon$, where Δ*ω*_*G*_ is the change in frequency with respect to the frequency of the equilibrium structure, ${\omega }_{G}^{0}$ is the frequency at the *G*-point for unstrained arsenene, and *ε* is the applied tensile strain. The Grüeinsen parameter shows the contribution to the change of thermal conductivity under strain and is summarized in Table S1. As can be seen, it increases with tensile strain, which shows the anharmonicity is strong and further indicates that the thermal conductivity will decrease. This behavior is different from that of graphene, where *γ* decreases with strain. However, as arsenene is compressed, the buckling also increases, which further decreases the Grüeinsen parameter.

The lattice vibrations of planar arsenene have also been calculated (see Fig. S2). Planar arsenene has negative frequencies in the whole lattice constant range. Though Fig. 1(b) and Table S1 show that planar arsenene is energetically more stable at larger lattice constants, the soft modes in the phonons indicate that planar arsenene is no longer stable, even at larger lattice constants where spontaneous buckling cannot be expected.

**Figure 4:**
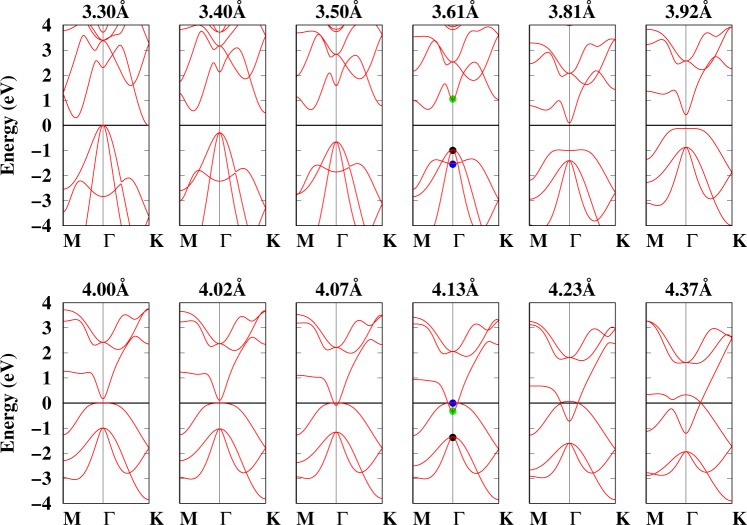
Band structures of buckled arsenene under different strains calculated without SOC. See the text for black, green, and blue symbols. The horizontal line shows the Fermi level, which is set to zero eV.

In the above section, we have shown that at the equilibrium geometry and small lattice constants buckled arsenene has negative frequencies, which were stabilized through tensile strain. To understand the atomic origin of instabilities/stabilities of buckled arsenene, the electronic structures are calculated. Figure 4 shows the electronic structures of buckled arsenene under different strains without SOC. The horizontal line shows the Fermi level, which is set to zero eV. First we shed light on the electronic band structure with zero strain. The band structure clearly shows that arsenene is an indirect bandgap semiconductor —the valence band maximum (VBM) lies at the Γ-point and the conduction band minimum (CBM) occurs along the Γ-*M*-direction. A direct band gap, where both the VBM and CBM lie at the Γ-point, can also be seen. The calculated indirect (direct) band gap is 1.61 (1.97) eV, which is in agreement with previous results^12,13,36,37^. We also calculated it with HSE and obtained for the indirect (direct) band gap a value of 2.89 (2.33) eV (see Fig. S4). The HSE calculated band gap also agrees with the previous work^13,14,52^. Two degenerate bands (marked with black dots)—mainly contributed by *p*_*x*_/*p*_*y*_-orbitals (see Fig. S5) at the Γ-point around 1 eV below the Fermi energy can be seen, as well as dispersive bands along the Γ-*K* or the Γ-*M*-directions. The *p*_*z*_-orbital (see Fig. S5) derived band, just a few meV below the degenerate bands (marked with blue dots), is also visible. These bands are very susceptible to external strains. The degenerate band at the Γ-point shifts towards the Fermi energy, and the *p*_*z*_-derived band moves to lower energies as the compressive strain is increased. For compressed arsenene, both indirect and direct band gaps exist.

For tensile strains, the *p*_*z*_-like band shifts toward the Fermi energy and disentangles from the heavy-hole bands, and the bands in the CB at the Γ-point (marked with green dots) also shift toward the Fermi energy. The *p*_*z*_ band touches the Fermi energy, so that arsenene behaves as a direct band gap semiconductor at 3.81 Å. The direct band gap decreases with tensile strain, and finally the band gap closes at 4.07 Å (13% strain). The band gap closes only at the Γ-point, but the band structure still has a finite electronic band gap at the M/K points, which indicates that strained arsenene has a semimetallic nature.The band closing at ~13% tensile strain indicates a possible signature of topological phase transition. At the topological transition point, the CBM and VBM touch each other at the Γ-point. For strains larger than 13%, the *p*_*z*_ band becomes partially occupied and a TI phase develops at 4.13 Å, where a clear band inversion can also be seen. The band inversion at the Γ-point and the opposite parity of the CBM and VBM prove that arsenene can therefore become a topological insulator under tensile strain. It is noted that in the TI phase of arsenene the Dirac cone also appears in the Brillouin zone along the Γ-K path and it is anisotropic in reciprocal space^34^.

**Figure 5:**
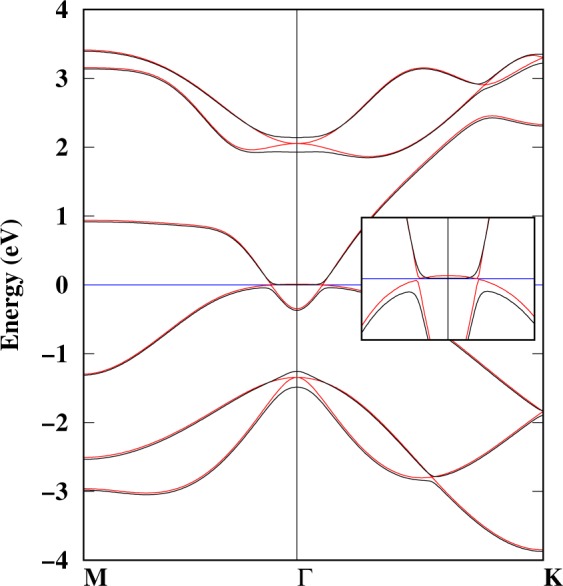
Electronic band structure of arsenene at 4.13 Å (14% strain) with and without -SOC. The black (red) lines correspond to a calculation with (without) SOC. The inset shows the band structure at the Γ-point between −1.0 and 1 eV.

Before we discuss the *Z*_2_ invariance, it is necessary to consider the effect of SOC on the electronic structure of arsenene under strain. The electronic structure calculated with SOC is shown in Fig. S3. The behavior of arsenene under strain with SOC is similar to that in Fig. 4, i.e. strain induces TI and the direct band gap decreases with tensile strain. However, with SOC the degeneracy of orbitals at the Γ-point is removed. Fig. S3 also shows that in the TI phase, the SOC opens a bandgap at the Dirac cone. The strength of the SOC is calculated to be ~270 meV, which is much larger than that of graphene and silicene^26^. To explicitly compare the effect of SOC on the electronic structure of arsenene under 14% strain (4.13 Å), Fig. 5 shows the electronic band structure calculated with and without SOC. The effect of SOC is clearly strong at the Γ-point. So at the VBM, the SOC increases the band gap. The calculated indirect band gap with (without) SOC is 43 (2.0) meV. The inset in Fig. 5 clearly highlights the effect of SOC. The band structures of planar arsenene is also calculated (see Fig. S6), but it does not show any significant signature that can be relevant for nano electronics, so we will not discuss it.

**Figure 6:**
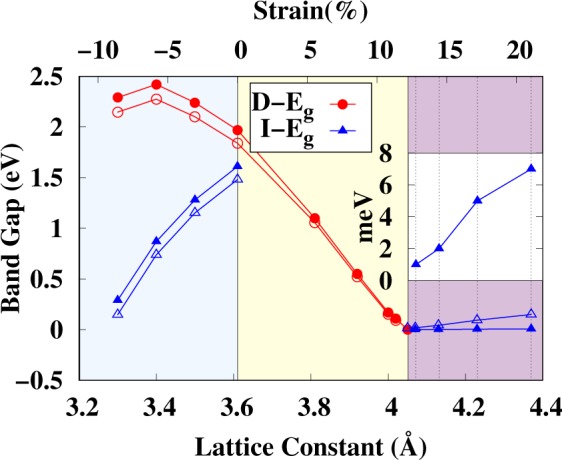
Band gap (eV) as function of the lattice constant (Å) (strain/%). Filled circles (triangles) show direct (indirect) band gap. The inset shows a zoomed bandgap (in meV). Open symbols correspond to the the band gap calculated with SOC.

From Figs 4 and S3 the direct and indirect band gaps are also calculated, which are summarized in Fig. 6. Figure 6 shows that arsenene has two band gaps, i.e., direct and indirect, when the lattice constant is ≤3.61 Å. In the region 3.20 Å ≤ *a* ≤ 3.61 Å, the direct (indirect) band gap increases (decreases) with increasing (compressing) the lattice strain. Note that this is the region where arsenene shows a dynamic instability in the phonons (see Fig. 3). Hence, the dynamic instability in arsenene under compressive strain could be related to the existence of two band gaps (direct and indirect). Whereas for 3.61 Å ≤ *a* ≤ 4.02 Å, arsenene only has a direct band gap, which decreases linearly with the lattice constant—again note that in this region arsenene is dynamically stable. Therefore, the absence of an indirect band gap in this region illustrates that arsenene is stabilized through the presence of a direct band gap, where electron momentum is conserved. When the lattice constant is larger than 4.05 Å, arsenene acquires a TI behaviour with a very small indirect band gap (in meV) which increases with strain. The SOC calculated band gap values are also shown in Fig. 6.

**Figure 7:**
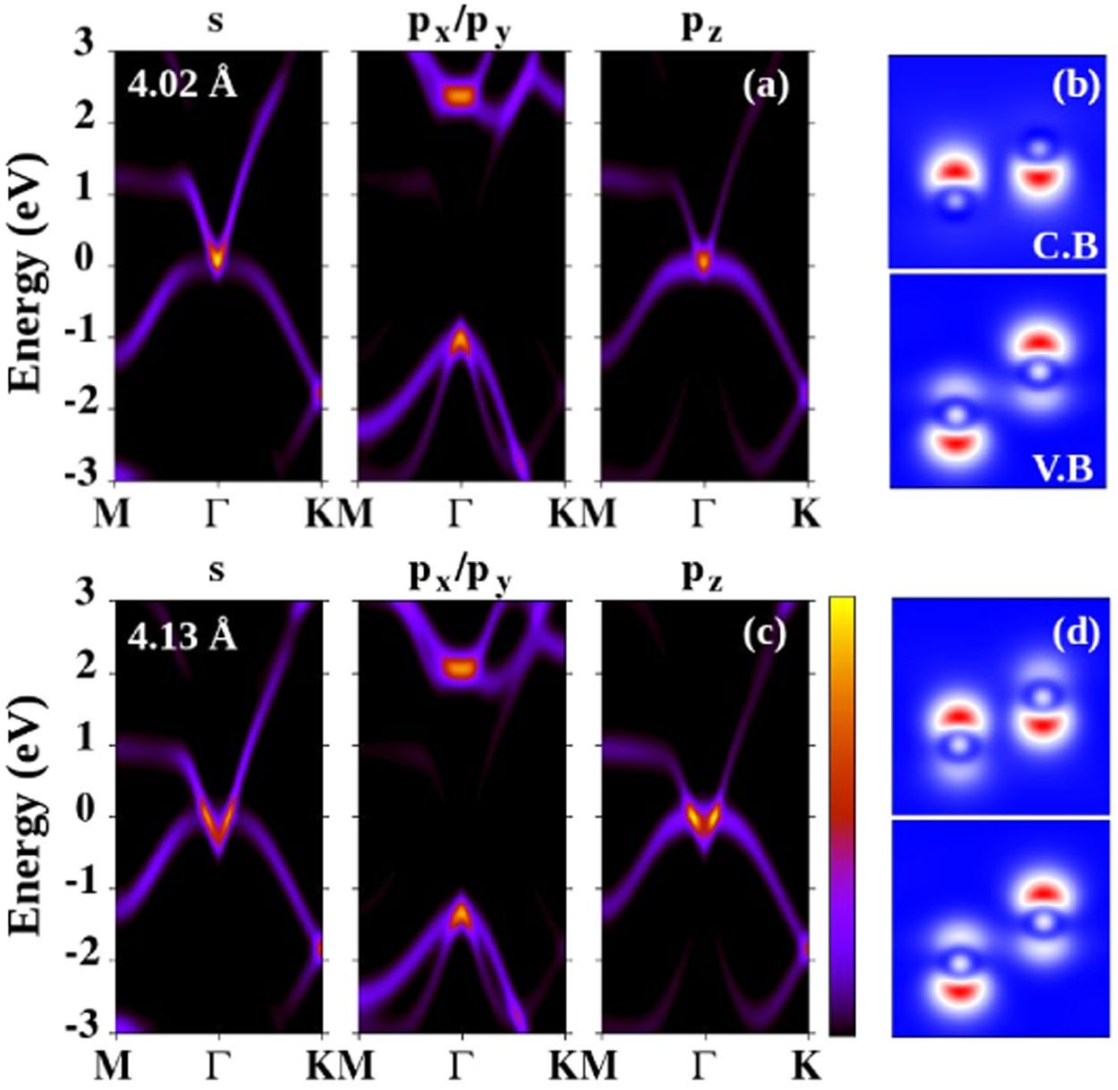
Orbital resolved band structure of buckled arsenene at (**a**) 4.02 Å, and (**c**) 4.13 Å. The charge densities near the conduction band (CB) and valence band (VB) are shown in (**b**,**d**).

To understand the origin of the band inversion and the TI phase, we show the orbital projected (*s*, *p*_*x*_, *p*_*y*_, *p*_*z*_) band structures calculated at 4.02 Å in Fig. 7(a) and 4.13 Å in Fig. 7(c). Figure 7(a) shows that the bands at the Fermi energy have *s* and *p*_*z*_ character. However, when arsenene is subjected to external tensile strain (see Fig. 7(c)) the *p*_*z*_-orbital driven band crosses the Fermi energy and the *s*-orbital derived band shifts below the Fermi energy, so that the band inversion takes place. Hence the band inversion is mainly contributed by the *p*_*z*_ and *s* electrons. The local charge densities near the Fermi energy in the conduction and valence bands are also analysed. In Fig. 7(b), the electrons have spherical-like symmetry in the conduction band, whereas they have *p*-electron character in the valence band. The symmetry of the orbitals changes when arsenene is strained (Fig. 7(d)) and in the VB and CB the distribution of electrons becomes *p*-like.

**Figure 8:**
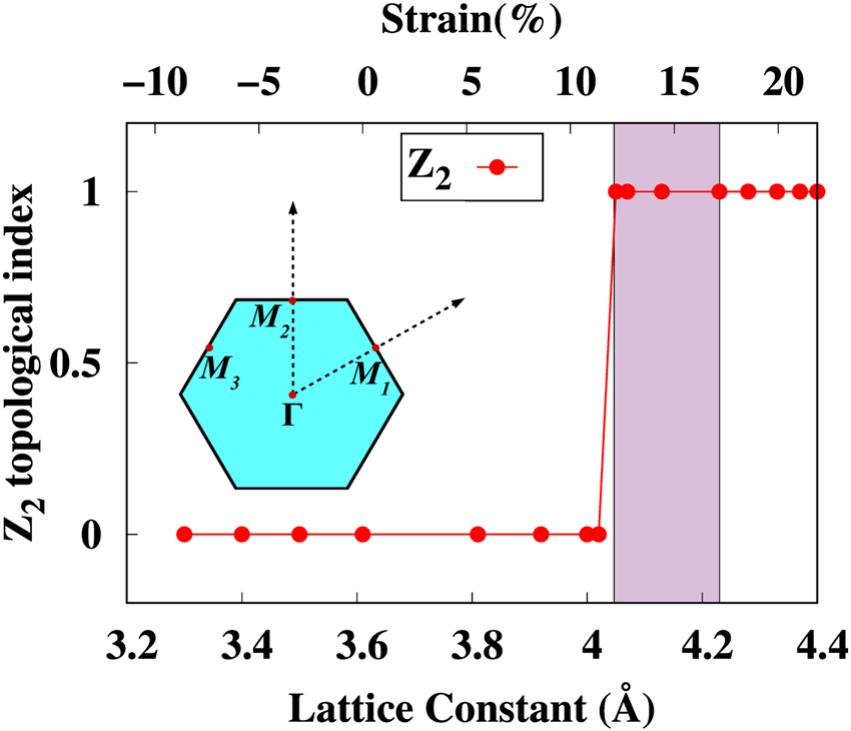
Calculated *Z*_2_ as a function of strain. The inset shows the TRIM-points in the BZ of arsenene. The violet color highlights the region where the TI phase of arsenene is dynamically stable.

Finally, the topological characteristics of the band structures of arsenene under strain are analysed by computing the *Z*_2_ topological invariant *ν* through the analysis of the parity of the wave functions at the time-reversal invariant momentum points (TRIM) in the 2D-BZ (see inset in Fig. 8). This procedure is applicable to crystals with inversion symmetry as proposed by Fu *et al*.^53^. If a system has centrosymmetry (also called inversion symmetry), a parity-based method can be employed to find the *Z*_2_ topological index^53^. The parity eigenvalue of occupied energy states is determined at the TRIM. We implemented the above method to prove the presence of a TI state in 2D monolayer arsenene tuned by biaxial strain. The *Z*_2_ invariant *ν* can be determined in terms of products of parity eigenvalues of Kramers doublets of occupied valence states at the four TRIM *K*-points (*K*_*i*_) as follows

1 $$\delta ({K}_{i})=\prod _{j=1}^{N}\,{\xi }_{2j}^{i}\,{\rm{and}}\,{(-1)}^{\nu }=\prod _{i=1}^{4}\,\delta ({K}_{i})$$

where $\delta ({K}_{i})$ is the product of parity eigenvalues at the TRIM points, *ξ*_2*j*_ = ±1 is the parity eigenvalue and the number of occupied bands is 2*N*. The value of *Z*_2_ invariant *ν* is calculated with SOC at every lattice constant (strain) and the results are summarized in Fig. 8. The parities of the orbitals are given in Table S2. At the equilibrium structure the parities of the orbitals at the four TRIM points are −, −, −, − implying that arsenene has a *Z*_2_ invariant *ν* = 0, i.e., that of a trivial insulator. At the topological transition point (4.02 Å), however, the conduction and valence states meet each other at the Γ-point. The parity of the orbitals in the valence band (near the Fermi energy) changes at 4.05–4.40 Å (the parities are +, −, −, −) and arsenene becomes a topologically non-trivial insulator with *Z*_2_ invariant *ν* = 1. So the parity of the orbital changes near the Fermi energy. Notice that arsenene shows a non trivial insulating behavior for *a* = 4.05–4.40 Å, where the band structures show a TI behavior (see Figs 4 and S7), however for lattice constants >4.23 Å (see Figs 3 and S7), buckled arsenene shows a dynamic instability. Figure 8 highlights the region with violet color where arsenene is dynamically stable with *Z*_2_ invariant *ν* = 1. Therefore, the TI phase of arsenene is dynamically stable for lattice constants <4.23 Å and can be a candidate material for QSH devices. This concludes that the role of phonons cannot be ignored when proposing a new material.

To summarize, we used density functional theory to investigate the non trivial and trivial insulating behaviors of arsenene under compressive and tensile strains. We found that buckling strongly depends on the lattice strain, which implies that each lattice constant determines its own buckling. The calculated cohesive energies show that planar arsenene is not stable as compared with buckled arsenene. The phonon calculations reveal that compressive strain destabilises arsenene, whereas at least 5% tensile strain is needed to dynamically stabilize arsenene. The optical bandgap between the optical and acoustic branches of phonon decreases with strain, and at 14% strain both branches hug each other. Arsenene remains stable under 18% strain, but larger strain destabilises it. The electronic band structure is found to have both direct and indirect bandgaps at the equilibrium lattice constant. However, under 5% tensile strain arsenene is found to be a direct bandgap semiconductor. The bandgap decreases with strain and closes at 13% strain. The bandgap closing produces a band inversion and a transition to a topological insulator behavior at 14% strain. The same conclusions were found by including the spin-orbit coupling in our calculations. Tensile strain helps to drive arsenene towards a topological insulating state, whereas the SOC opens a band gap near the Fermi energy. The band inversion was discussed using the orbital projected band structure and the charge densities near the conduction and valence band regions. Finally, the topological characteristics of the band structures of arsenene under strain were analysed by computing the *Z*_2_ topological invariant *ν* through the analysis of the parity of the wave functions at the time-reversal invariant momentum points in the 2D-BZ. We showed that the topological invariant *ν* is 1 for arsenene under 14% tensile strain, which implies that arsenene has a non-trivial insulating behavior. Phonons are used as a tool to address the dynamic stability of the topological phase of arsenene. The dynamically stable monolayer can be a candicate material for nano electronic devices where a large electrical conductivity is required.

## Methods

First-principles calculations based on density functional theory (DFT) were performed with the plane-wave and pseudopotential method implemented in the Quantum Espresso package^54^. The exchange and correlation energy and potential were calculated with the Perdew-Burke-Ernzerhof (PBE) parametrization^55^ of the generalized gradient approximation (GGA). The ultrasoft pseudopotentials were parameterized with the recipe of Rappe, Rabe, Kaxiras and Joannopoulos^56^. For the spin-orbit coupling (SOC), the core electrons were treated fully relativistically. The electron wave function was expanded in a plane wave basis set cut-off of 30 Ry. A dense 40 × 40 × 1 Monckhorst-Pack grid^57^ was used for the *k*-points, which gave a fine reciprocal-space grid and hence a rather high accuracy. A vacuum slab of 15 Å was used in the direction normal to the plane of arsenene to ensure the absence of interlayer interactions in that direction. The convergence of all computational parameters was checked carefully. The phonon frequencies were computed using density-functional perturbation theory (DFPT) with a 6 × 6 × 1 *q*-point mesh.Test calculations with the Heyd-Scuseria-Ernzerhof (HSE06)^58^ hybrid functional were also employed to compute more correct bandgaps and check the robustness of our study. We will only present our GGA data and comparison with HSE will be addressed wherever is required.

## Supplementary information

Supplementary information

## Data Availability

There are no restrictions on data availability.
