# Supplementary material for: Dynamically Stable Topological Phase of Arsenene
Source: Sci Rep. 2019 May 28;9:7966. doi: 10.1038/s41598-019-44444-4 (PMC6538655; doi:10.1038/s41598-019-44444-4)
Supplement: Supplementary file 1 — Supplementary information [file 41598_2019_44444_MOESM1_ESM.pdf]

Supplementary Information for:
Dynamically Stable Topological Phase of Arsenene

Gul Rahman* and Asad Mahmood

Department of Physics, Quaid-i-Azam University, Islamabad 45320, Pakistan

Víctor M. García-Suárez

Departamento de Física, Universidad de Oviedo, 33007 Oviedo Spain and
Nanomaterials and Nanotechnology Research Center (CINN), Spain

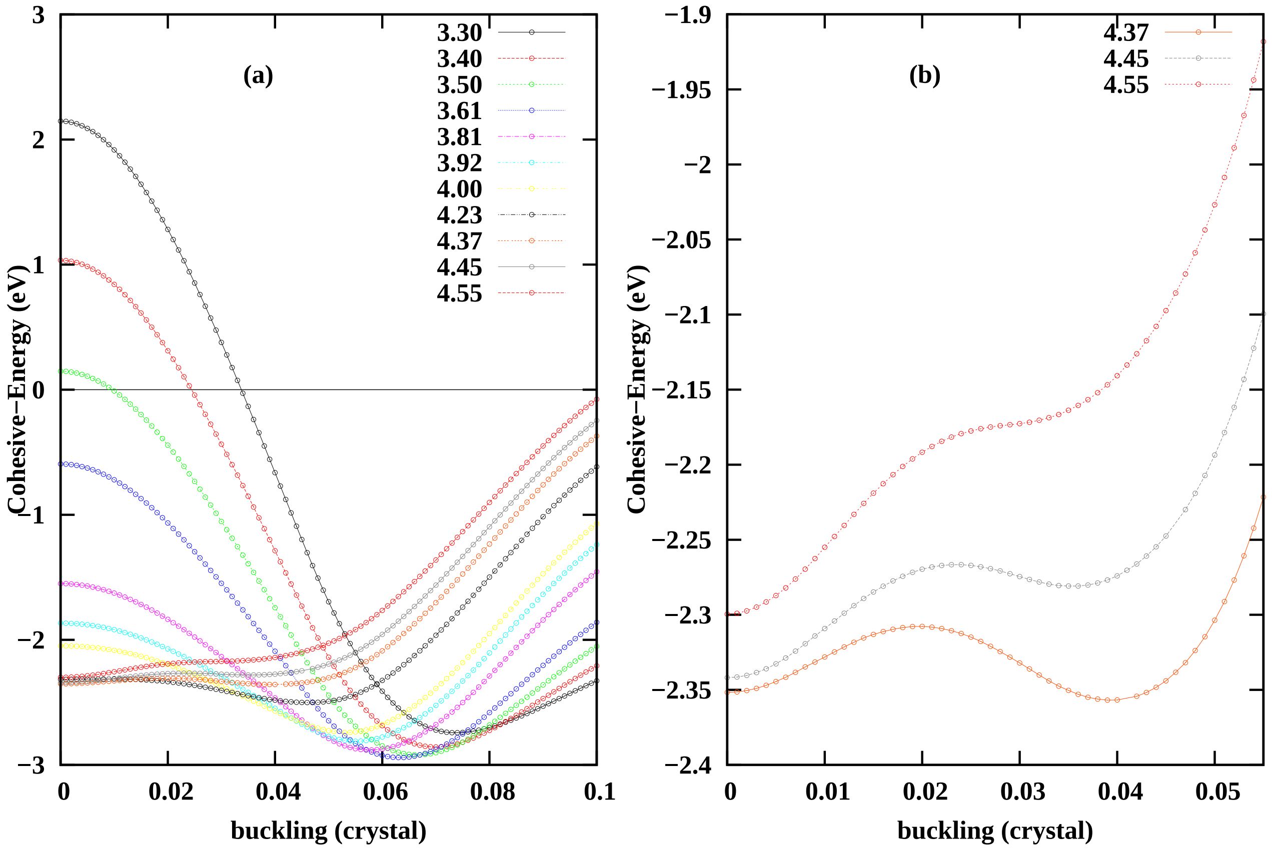

Fig.S 1: (a) Calculated cohesive energy (in eV) vs buckling (in crystal coordinates) for different lattice constants (in Å) (b) The same as in (a) but for 4.37Å, 4.45Å, and 4.55Å. The solid points are calculated data points.

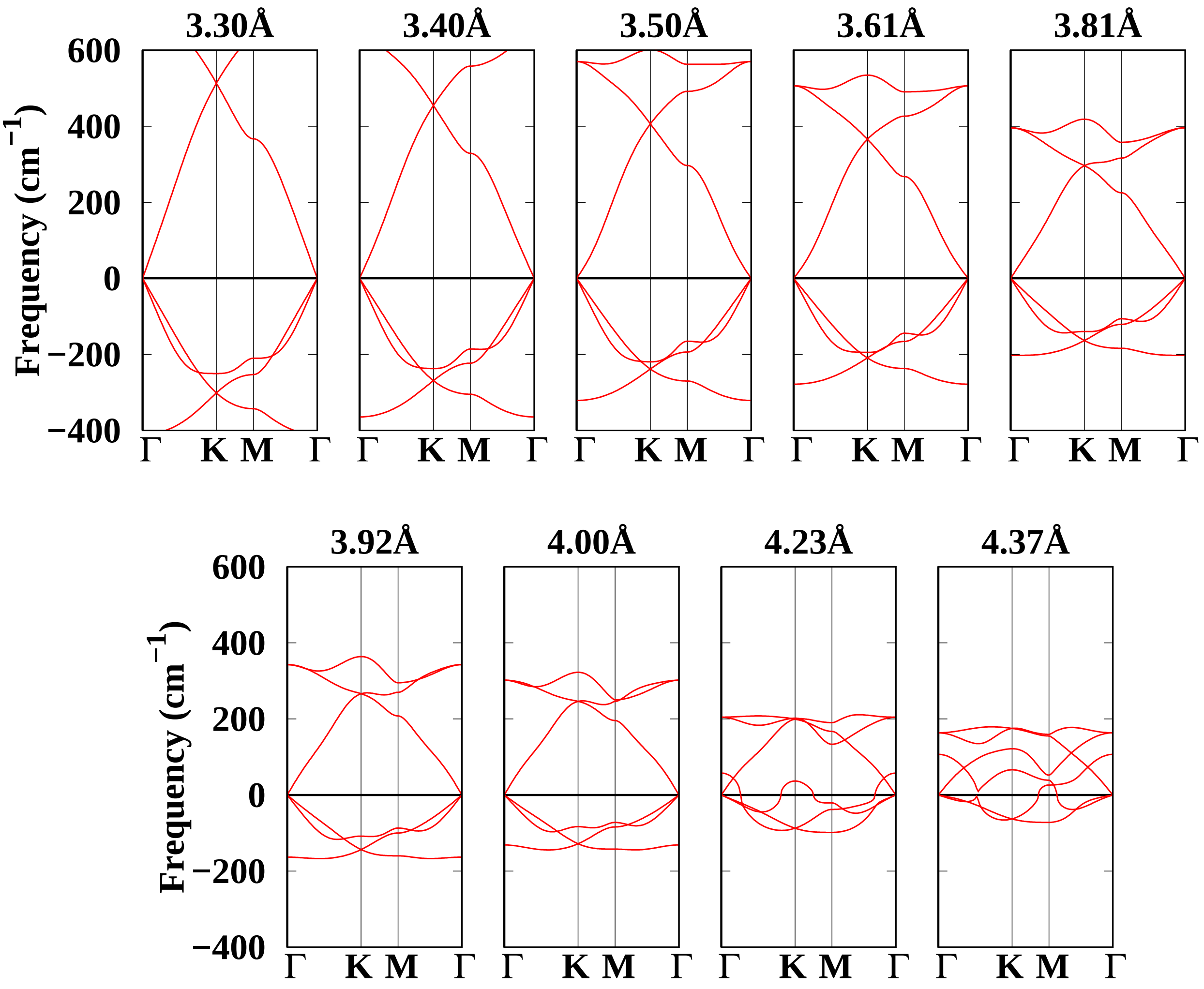

Fig.S 2: Calculated phonon dispersion curves of planar arsenene under different strains.

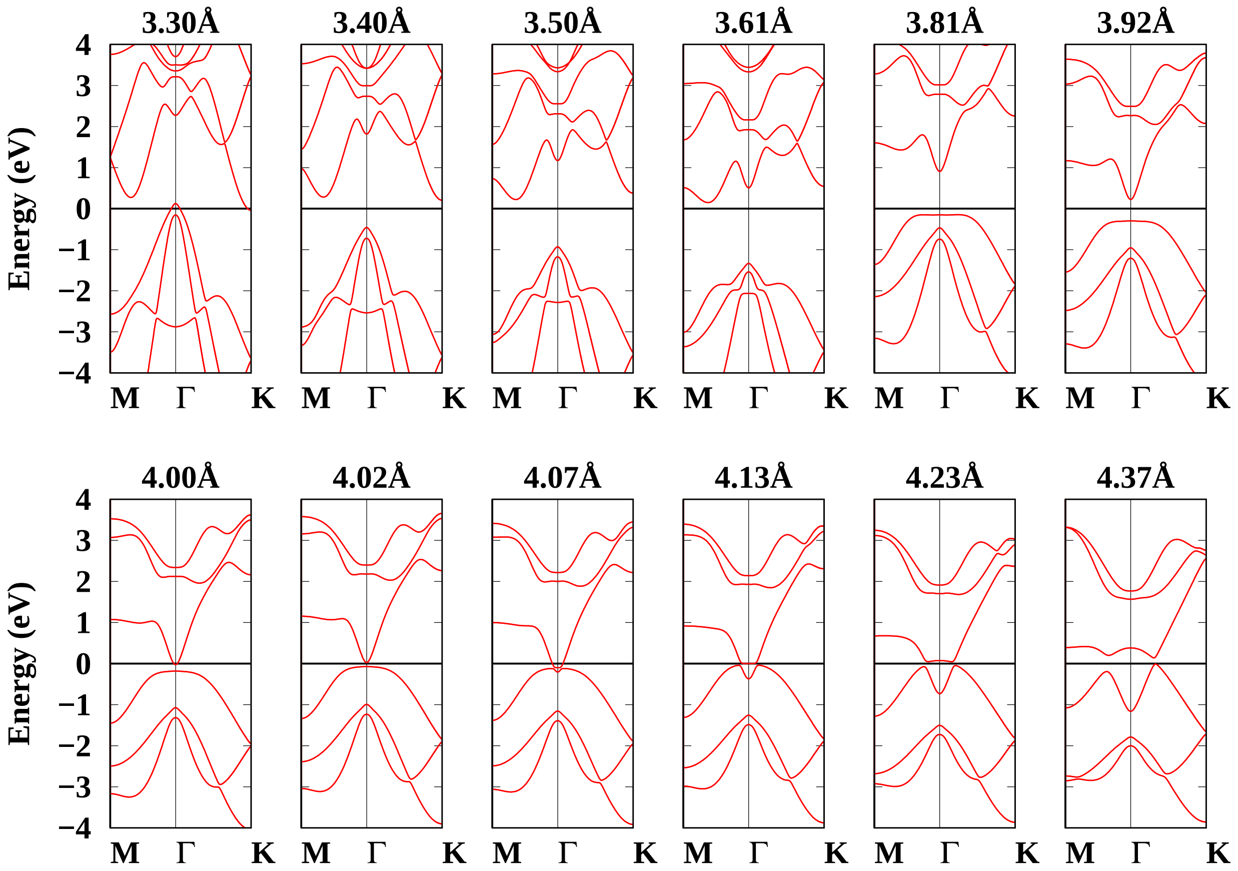

Fig.S 3: Calculated band structures of buckled arsenene under different strains calculated with SOC. The horizontal line shows the Fermi level which is set to zero eV.

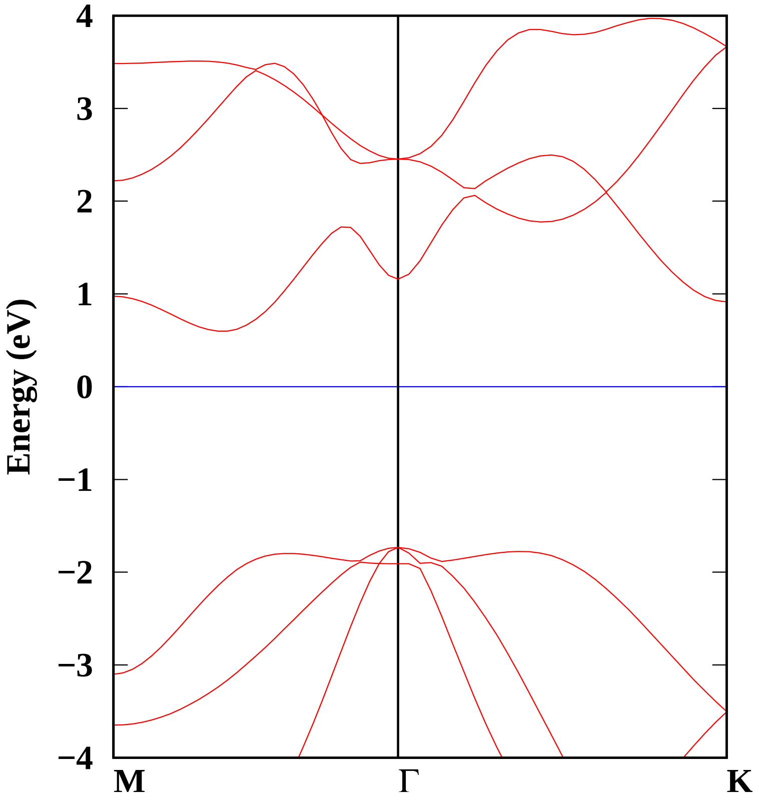

Fig.S 4: HSE calculated band structures of buckled arsenene. The horizontal line shows the Fermi level which is set to zero eV.

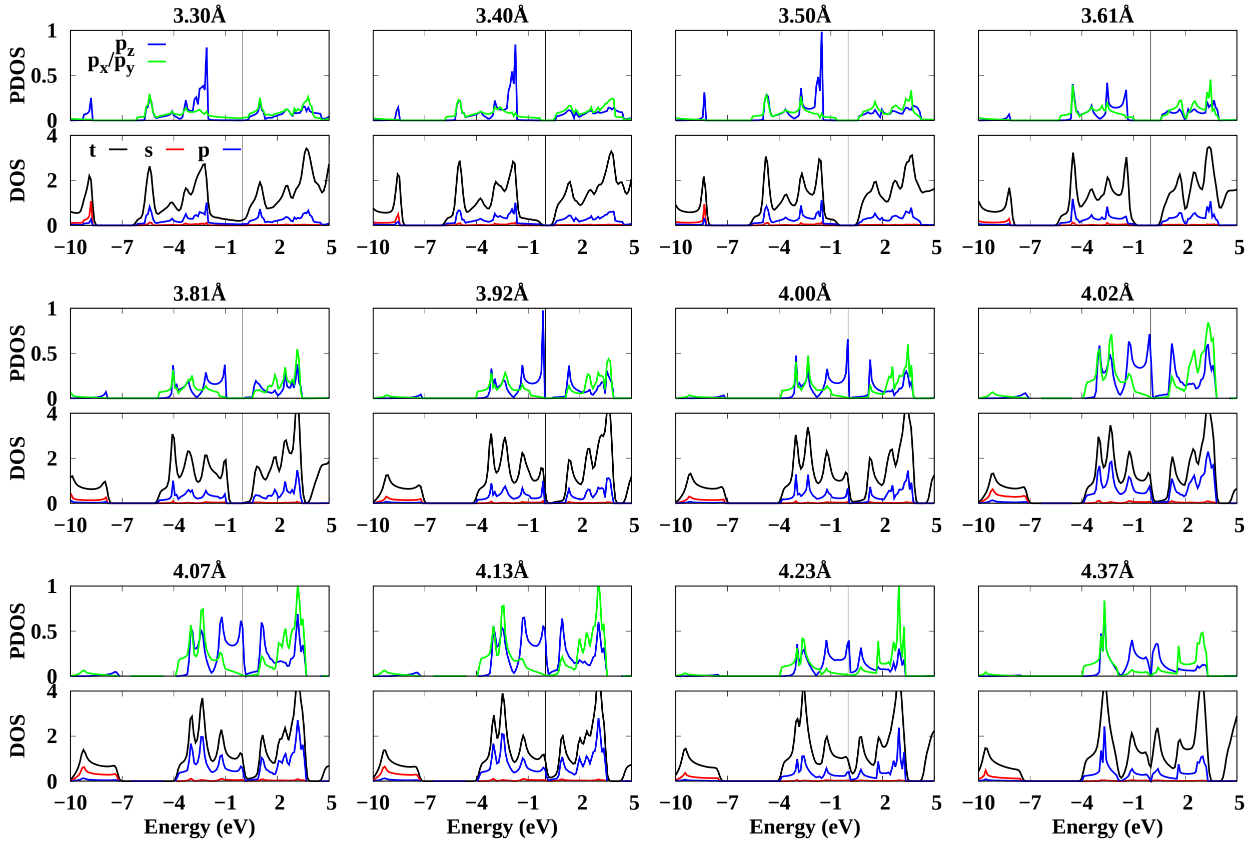

Fig.S 5: Calculated total (black) and orbitals (s,p) projected densities of states of buckled arsenene under differnet strains. The vertical line shows the Fermi level which is set to zero eV.

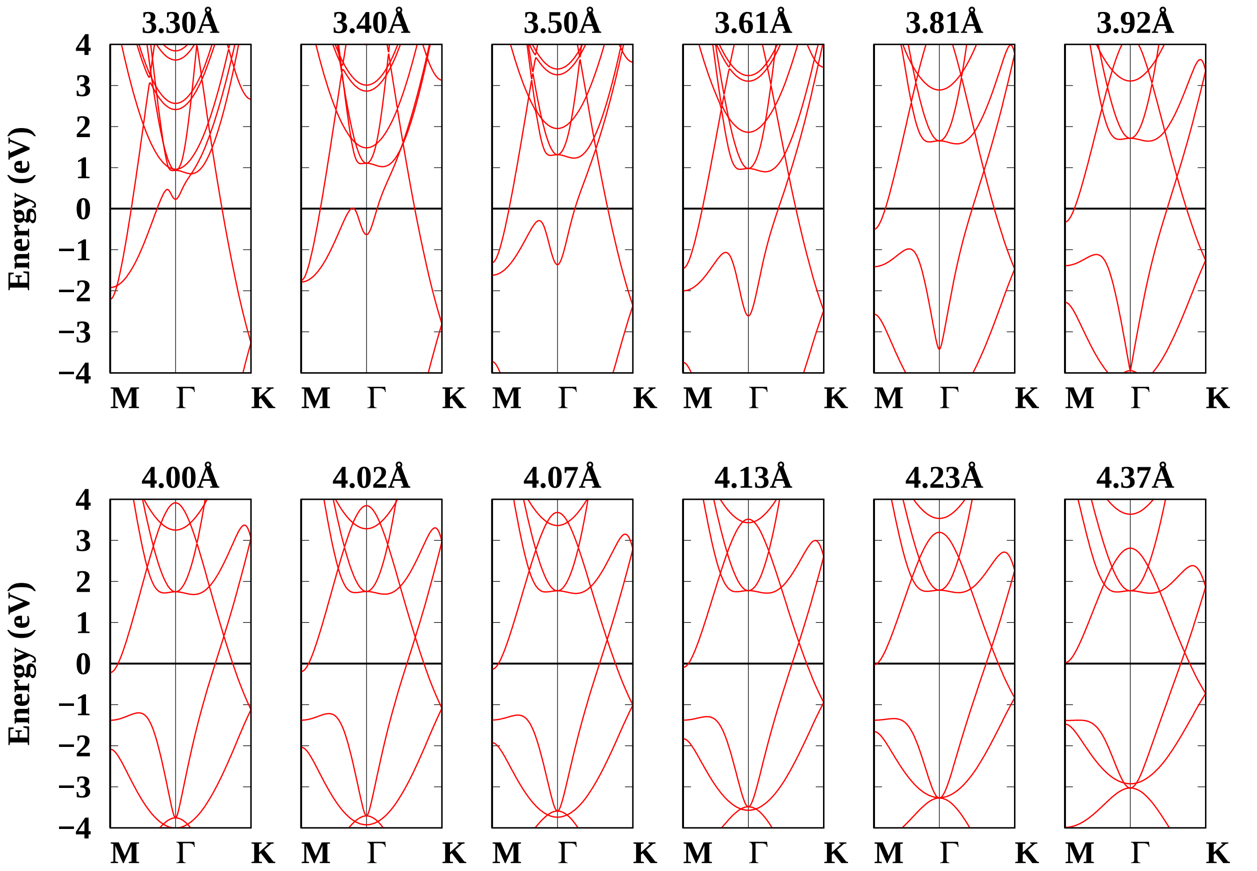

Fig.S 6: Calculated band structures of planar arsenene under different strains calculated without SOC. The horizontal line shows the Fermi level which is set to zero eV.

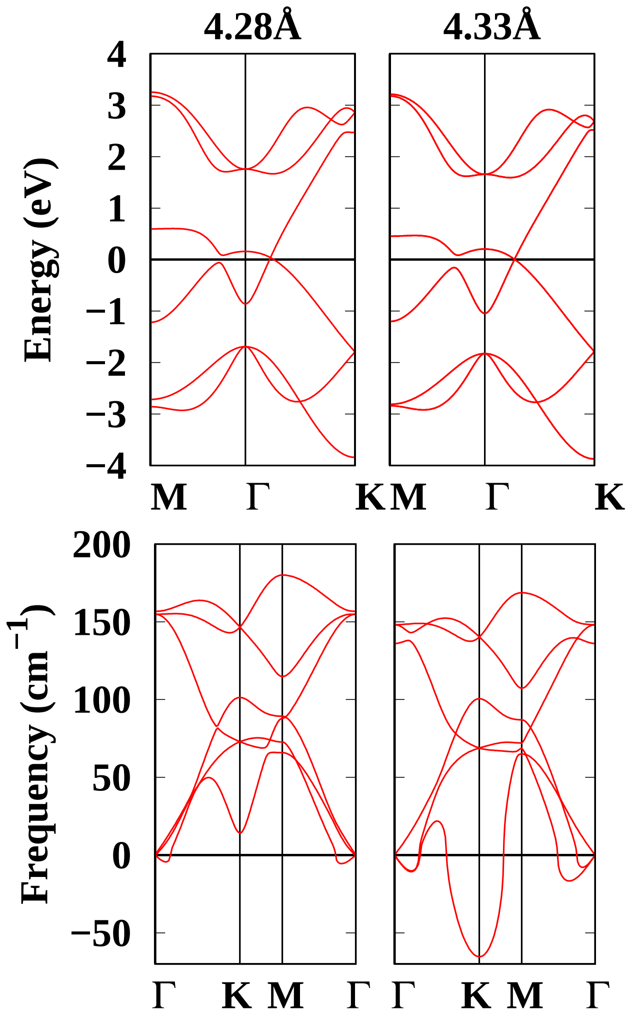

Fig.S 7: The upper panels show the calculated band structures (without SOC) of buckled arsenene for 4.28Å(left), and 4.33Å(right), where the horizontal line shows the Fermi level which is set to zero eV. The lower panels show the calculated phonons of buckled arsenene for 4.28Å(left), and 4.33Å(right).

Table.S I: Optimized lattice parameter a (in Å),buckling height Δ (in Å), bond length l (in Å), bond angle θ (in degree), band gap E_g (in eV), cohesive energy E_c (in eV/atom), G & D points (in cm^{-1}) and Gruneisen parameter (γ_G) of buckled arsenene monolayer. E_g is calculated for indirect(direct) band gap, while values written in parentheses for E_c represent planar arsenene monolayer calculations. Values in the bracket in the first column shows strain.

a	Δ	l	θ	E_g	E_c	G	D	γ_G
3.30(-8.52)	1.49	2.42	86.01	0.29(2.29)	-2.74 (2.15)	302	262	-0.06
3.40(-5.73)	1.45	2.44	88.29	0.87(2.42)	-2.86 (1.03)	326	256	0.60
3.50(-2.94)	1.42	2.47	90.19	1.28(2.24)	-2.92 (0.15)	316	244	0.65
3.61(0.00)	1.39	2.51	92.05	1.61(1.97)	-2.94 (-0.59)	305	229	—
3.81(5.73)	1.34	2.58	95.27	—(1.10)	-2.88 (-1.55)	284	203	0.59
3.92(8.66)	1.32	2.62	96.85	—(0.55)	-2.81 (-1.87)	269	188	0.68
4.00(10.9)	1.30	2.65	98.02	—(0.17)	-2.74 (-2.05)	249	178	0.84
4.02(11.6)	1.30	2.66	98.20	—(0.11)	-2.72 (-2.09)	245	175	0.85
4.05(12.3)	1.28	2.67	98.76	—(0.00)	-2.69 (-2.13)	237	172	0.90
4.07(13.1)	1.28	2.68	98.96	0.001(—)	-2.67 (-2.17)	231	168	0.92
4.13(14.5)	1.25	2.69	100.96	0.002(—)	-2.61 (-2.23)	215	163	1.02
4.23(17.5)	1.19	2.72	102.20	0.005(—)	-2.50 (-2.32)	177	152	1.20
4.37(21.3)	1.05	2.73	106.15	0.007(—)	-2.36 (-2.35)	142	135	1.26

Table.S II: Parities of all occupied bands at the four TRIM points (Γ, M_1, M_2, M_3) in BZ for different lattice constants a (in Å). Values in the brackets, e.g. (+) shows the parity at TRIM point. The last column show the Z_2 invariant ν values.

a	$\Gamma(0,0,)$	$M_1(0.5,0)$	$M_2(0,0.5)$	$M_3(0.5,0.5)$	Z_2
3.30	$+-+++(-)$	$-++--(-)$	$-++--(-)$	$-++--(-)$	0
3.40	$+-+++(-)$	$-++--(-)$	$-++--(-)$	$-++--(-)$	0
3.50	$+-+++(-)$	$-++--(-)$	$-++--(-)$	$-++--(-)$	0
3.61	$+-+++(-)$	$-++--(-)$	$-++--(-)$	$-++--(-)$	0
3.81	$+-+++(-)$	$-++--(-)$	$-++--(-)$	$-++--(-)$	0
3.92	$+-+++(-)$	$-++--(-)$	$-++--(-)$	$-++--(-)$	0
4.00	$+-+++(-)$	$-++--(-)$	$-++--(-)$	$-++--(-)$	0
4.02	$+-+++(-)$	$-++--(-)$	$-++--(-)$	$-++--(-)$	0
4.05	$+-++-(+)$	$-++--(-)$	$-++--(-)$	$-++--(-)$	1
4.07	$+-++-(+)$	$-++--(-)$	$-++--(-)$	$-++--(-)$	1
4.13	$+-++-(+)$	$-++--(-)$	$-++--(-)$	$-++--(-)$	1
4.23	$+-++-(+)$	$-++--(-)$	$-++--(-)$	$-++--(-)$	1
4.28	$+-++-(+)$	$-++--(-)$	$-++--(-)$	$-++--(-)$	1
4.33	$+-++-(+)$	$-++--(-)$	$-++--(-)$	$-++--(-)$	1
4.37	$+-++-(+)$	$-++--(-)$	$-++--(-)$	$-++--(-)$	1
4.40	$+-++-(+)$	$-++--(-)$	$-++--(-)$	$-++--(-)$	1

* Electronic address: gulrahman@qau.edu.pk
